# Physicians’ Attitudes and Use of E-Cigarettes as Cessation Devices, North Carolina, 2013

**DOI:** 10.1371/journal.pone.0103462

**Published:** 2014-07-29

**Authors:** Kelly L. Kandra, Leah M. Ranney, Joseph G. L. Lee, Adam O. Goldstein

**Affiliations:** 1 Psychology Department, Benedictine University, Lisle, Illinois, United States of America; 2 Department of Family Medicine, University of North Carolina, Chapel Hill, North Carolina, United States of America; 3 Departments of Family Medicine and Health Behavior, University of North Carolina, Chapel Hill, North Carolina, United States of America; The University of Auckland, New Zealand

## Abstract

**Introduction:**

Electronic cigarettes (e-cigarettes) are not currently approved or recommended by the Food and Drug Administration (FDA) or various medical organizations; yet, they appear to play a substantial role in tobacco users’ cessation attempts. This study reports on a physician survey that measured beliefs, attitudes, and behavior related to e-cigarettes and smoking cessation. To our knowledge this is the first study to measure attitudes toward e-cigarettes among physicians treating adult smokers.

**Methods:**

Using a direct marketing company, a random sample of 787 North Carolina physicians were contacted in 2013 through email, with 413 opening the email and 128 responding (response rate = 31%). Physicians’ attitudes towards e-cigarettes were measured through a series of close-ended questions. Recommending e-cigarettes to patients served as the outcome variable for a logistic regression analysis.

**Results:**

Two thirds (67%) of the surveyed physicians indicated e-cigarettes are a helpful aid for smoking cessation, and 35% recommended them to their patients. Physicians were more likely to recommend e-cigarettes when their patients asked about them or when the physician believed e-cigarettes were safer than smoking standard cigarettes.

**Conclusions:**

Many North Carolina physicians are having conversations about e-cigarettes with their patients, and some are recommending them. Future FDA regulation of e-cigarettes may help provide evidence-based guidance to physicians about e-cigarettes and will help ensure that patients receive evidence-based recommendations about the safety and efficacy of e-cigarettes in tobacco cessation.

## Introduction

The 2008 Treating Tobacco Use and Dependence Clinical Practice Guideline recommends that clinicians ask all patients about tobacco use, offer strong cessation messages, and provide assistance to those patients who use tobacco [1]. Recommended treatments for tobacco cessation include counseling and/or medications such as Bupropion SR or nicotine replacement (e.g., nicotine patch, gum, or inhaler). The combination of behavioral counseling with pharmacotherapy is also strongly recommended [1]. These guidelines do not discuss the use of electronic cigarettes (e-cigarettes), as the guidelines were written before e-cigarettes were widely available in the U.S. Since then, however, e-cigarettes have become a cessation tool for some tobacco users’ cessation attempts [2], despite their use not being approved or recommended by the FDA [3] or various medical organizations, including the American Lung Association [4], the American Medical Association [5]–[6], the American Thoracic Society [7], and the Center for Public Health and Tobacco Policy [8]. The purpose of the current study is to report on a physician survey that measured beliefs, attitudes, and behavior related to e-cigarettes as a tool for smoking cessation. To our knowledge, only one study thus far has sought to measure e-cigarettes from the perspective of physicians, and that study focused on adolescent providers [9]–[10]. This study is unique in that it measures e-cigarettes from the perspective of physicians who treat adult patients.

## Methods

### Ethics Statement

- This submission was reviewed by the UNC Biomedical IRB and Office of Human Research Ethics, which has determined that this submission does not constitute human subjects research as defined under federal regulations [45 CFR 46.102 (d or f) and 21 CFR 56.102(c)(e)(l)] and does not require IRB approval.
- This study was deemed as non-human subjects research, which is similar to an exemption. As a result, federal regulations for consent are not applicable and a waiver for participation was not required from participants.

### Recruitment and Sample

A random sample of North Carolina (NC) physicians were recruited to participate. From July–August, 2013, Infocus Marketing, Inc., a direct marketing company with access to the American Medical Association mailing list, attempted to contact 156 family medicine physicians, 161 internal medicine physicians, 159 obstetricians/gynecologists, 160 psychiatrists, and 151 surgeons (total recruitment, 787 providers) through three different waves of emails. From these emails, which invited physicians to participate in a survey on attitudes and use of QuitlineNC services for patients who use tobacco, 14 addresses were invalid or emails returned, 413 were opened, and 128 responded (28 family medicine physicians, 24 internal medicine physicians, 21 obstetricians/gynecologists, 27 psychiatrists, and 28 surgeons) for an overall response rate of 31%. Physicians were offered a $100 gift card as an incentive for participation, and every physician contacted had the opportunity to decline participation by unsubscribing from the survey. Physicians were assured their responses would remain anonymous.

### Survey Measures

A series of close-ended questions measured physicians’ attitudes towards e-cigarettes. Specifically, physicians were asked if they believe e-cigarettes are approved by the FDA for smoking cessation; if they believe e-cigarettes lower the risk of cancer for patients who use them instead of smoking cigarettes; if they believe e-cigarettes are a helpful aid for smoking cessation; and if they recommend use of e-cigarettes to their patients. Response options provided were *yes* and *no*. Physicians were also asked how often their tobacco-using patients ask about e-cigarettes, with response options given as *frequently*, *sometimes*, *rarely*, and *never*. In addition, the survey contained items measuring personal and professional demographics (e.g., gender, age, years in practice, specialty), as well as items measuring clinic behaviors and attitudes (e.g., how often they document counseling in clinic notes after offering tobacco use treatment to their patients and how confident they are in their ability to prescribe optimal doses of tobacco cessation medications). Physicians rated these items using a 4-point response scale with varying labels such as *most times* to *never* and *strongly agree* to *strongly disagree*.

### Analysis

Data were analyzed using SPSS version 21. Missing data were excluded from analysis, as were physicians who are not actively involved in clinical practice (*n* = 6). A positive response to recommending e-cigarettes to patients served as the outcome variable for a backward stepwise logistic regression analysis. After conducting a series of bivariate analyses, response categories were collapsed into two categories to ensure an adequate sample size within each category, and the following variables served as predictors: *agreement* with being extremely confident in ability to prescribe optimal doses (*disagreement* served as reference group); those who offer intensive counseling to those who use tobacco *most/sometimes* (*rarely* served as reference group); those who document counseling in clinic notes *most times* (*sometimes/rarely* served as reference group); *psychiatry* specialty (*others* served as reference group); *45 and older* (*44 and younger* served as reference group); frequency of patients asking about e-cigarettes (left as continuous); and *agreement* that e-cigarettes lower the risk of cancer for patients who use them instead of smoking cigarettes (*no* served as reference group). All variables used in the analysis may be found in Dataset S1. Nonstatistically significant predictors were removed from the model so that the final model included only those variables statistically significant at p<.05.

## Results

### Demographics

Of the *n* = 122 physicians who were active in clinical practice, 64.7% had 10 or more years in their field, 85.2% saw 26 or more patients in a typical week, and 56.6% lived in towns with a population greater than 100,000. In addition, a majority of physicians were male, white, and had never been smokers. Group settings accounted for 36.7% of the sample; however, many physicians practiced in a hospital or academic setting, 24.2% and 21.1%, respectively.

### E-cigarettes in Clinical Practice

Over two-thirds (67.2%) of the physicians indicated that e-cigarettes are a helpful aid for smoking cessation, and 35.2% recommended them to their patients. A majority (64.8%) believed that e-cigarettes lower the risk of cancer for patients who use them instead of smoking cigarettes. E-cigarettes were also frequently part of the clinical encounter, with 48.4% of physicians responding that patients ask about e-cigarettes frequently or sometimes. Only 20.5% of physicians indicated they are never asked about e-cigarettes. 13% of physicians incorrectly believed that e-cigarettes are already approved by the FDA for smoking cessation.

### Predictors of Recommending E-cigarettes

Table 1 presents the breakdown of variables included in the logistic regression model, and Table 2 presents the statistically significant logistic regression coefficients and odds ratios for predictors that remained in the final model. Increased odds of recommending e-cigarettes to patients is associated with physicians who believed e-cigarettes lower the risk of cancer for patients who use them instead of smoking cigarettes, increased frequency of patient inquiry about e-cigarettes, older physicians, and those physicians who documented tobacco use counseling in their clinic notes.

**Table 1:** Variables Included in Logistic Regression, 2013, n = 122.

Physician Characteristics	%
Extremely confident in ability to prescribe optimal doses	
Agree	62.8%
Disagree	37.2%
Offer intensive tobacco treatment counseling	
Most/Sometimes	63.6%
Rarely/Never	36.4%
Document counseling in clinic notes	
Most times	57.6%
Sometimes/rarely	42.4%
Specialty	
Psychiatry	21.1%
Other	78.9%
Age	
44 and younger	47.9%
45 and older	52.1%
Frequency patients ask about e-cigarettes*	
Frequently	20.5%
Sometimes	31.1%
Rarely	36.1%
Never	12.3%
Believe e-cigarettes lower risk of cancer	
Yes	64.8%
No	35.2%

*Variable treated as continuous.

**Table 2:** Significant Predictors of Recommending E-cigarettes, 2013, n = 122.

Significant Variables	Df	Sig.	Exp(B)
Provider Age (Reference = younger)	1	.021	3.110
Belief that e-cigarettes lower the risk of cancer	1	.001	6.817
Frequency which patients ask about e-cigarettes	1	.001	2.468
Physicians who document tobacco treatment counseling	1	.022	3.316

## Conclusions

### Principal findings

Previous reviews have found that e-cigarettes are viewed by the general public as effective strategies for quitting and reducing harm, [9] and research suggests some smokers use e-cigarettes for cessation purposes [11]. The question remains of whether physicians share those same attitudes regarding e-cigarettes.

To date, only one study of adolescent providers has sought to answer this question [9–10–12], and this research suggests that physicians who treat adolescents lack professional education when it comes to e-cigarettes and often learn about e-cigarettes directly from their patients [10]. In our study, approximately four out of five participating physicians reported being asked about e-cigarettes from their patients who used tobacco. Interest in e-cigarettes appears high, and, despite an absence of evidence regarding the long-term health impact of e-cigarettes [13], over one-third of physicians in this sample reported recommending their use for patients, and over two-thirds believed e-cigarettes are a helpful aid for smoking cessation. Although some evidence suggests e-cigarettes can be effective for cessation [2]–[14], they are not included in current guidelines that recommend combination nicotine replacement therapy or varenicline as first-line therapy [15]. Because current smokers who have tried e-cigarettes do not report an increased intention to quit smoking [16] and concerns exist over dual use of these products [17], physicians should remain cautious until more data is available about recommending e-cigarettes as tobacco cessation tools in clinical practice in favor of more effective modalities. Behavioral counseling about tobacco use cessation should also remain prominent in all quit attempts [1]. Furthermore, there is insufficient research on the relationship between e-cigarettes and nicotine dependence, including whether or not e-cigarettes could actually increase dependence [13]. To what extent e-cigarettes work more or less effectively than FDA approved pharmacotherapy remains unclear.

Our results also suggest that physicians who document counseling in their clinic notes after offering tobacco use treatment to their patients are more likely to recommend e-cigarettes. This relationship suggests that physicians may be interested in continuing the e-cigarette conversation with their patients in future appointments, as advising patients to quit smoking is the most often utilized intervention by physicians [18]. However, it is then imperative that physicians stay current with evidence-based research on e-cigarettes because discrepancies already exist among physicians when it comes to tobacco use treatment options [19]. Our results are no different in that older physicians were more likely to recommend e-cigarettes than younger physicians, and some physicians incorrectly believed they are already approved by the FDA for smoking cessation. Without widespread dissemination of clear, evidence-based research on e-cigarettes, it is likely these discrepancies will continue and patients could potentially be given inaccurate information [10].

### Limitations

This research has several limitations. As results are specific to a small sample of NC physicians, they may not generalize to other populations. Also, the response rate is relatively low and there is the potential for nonresponse bias. It is possible that our sample includes physicians who are more positive towards e-cigarettes than other non-participating physicians. However, our sample was recruited for a survey on the North Carolina Quitline without any indication there would be questions related to attitudes or behaviors regarding e-cigarettes as cessation devices. Furthermore, 31% for physicians participating in an email survey can be considered quite good [20–21–22]. Finally, results are descriptive in nature. Causality and directionality should not be inferred. Given the preliminary nature of this survey, it is recommended that ongoing surveillance of e-cigarettes as a tobacco use treatment option continues with a much larger, diverse, random sample of physicians.

### Conclusion

This research provides a first look at how e-cigarettes are being used as cessation devices among physicians who treat adult patients. Our results suggest that physicians see potential in these products as a cessation device and that some make recommendations for their use. As e-cigarettes become more mainstream, physicians may be called on to engage in conversations with their patients about the safety and efficacy of these products. It is essential that the FDA critically review the current evidence on e-cigarettes and provide clear guidance about e-cigarettes and tobacco cessation.

## Supporting Information

Dataset S1 (XLSX)
